# Supplementary material for: A fat-tissue sensor couples growth to oxygen availability by remotely controlling insulin secretion
Source: Nat Commun. 2019 Apr 26;10:1955. doi: 10.1038/s41467-019-09943-y (PMC6486587; doi:10.1038/s41467-019-09943-y)
Supplement: Supplementary file 1 — Supplementary Information [file 41467_2019_9943_MOESM1_ESM.pdf]

Supplementary information

A fat-tissue sensor couples growth to oxygen availability by remotely controlling insulin secretion

Texada, *et al.*

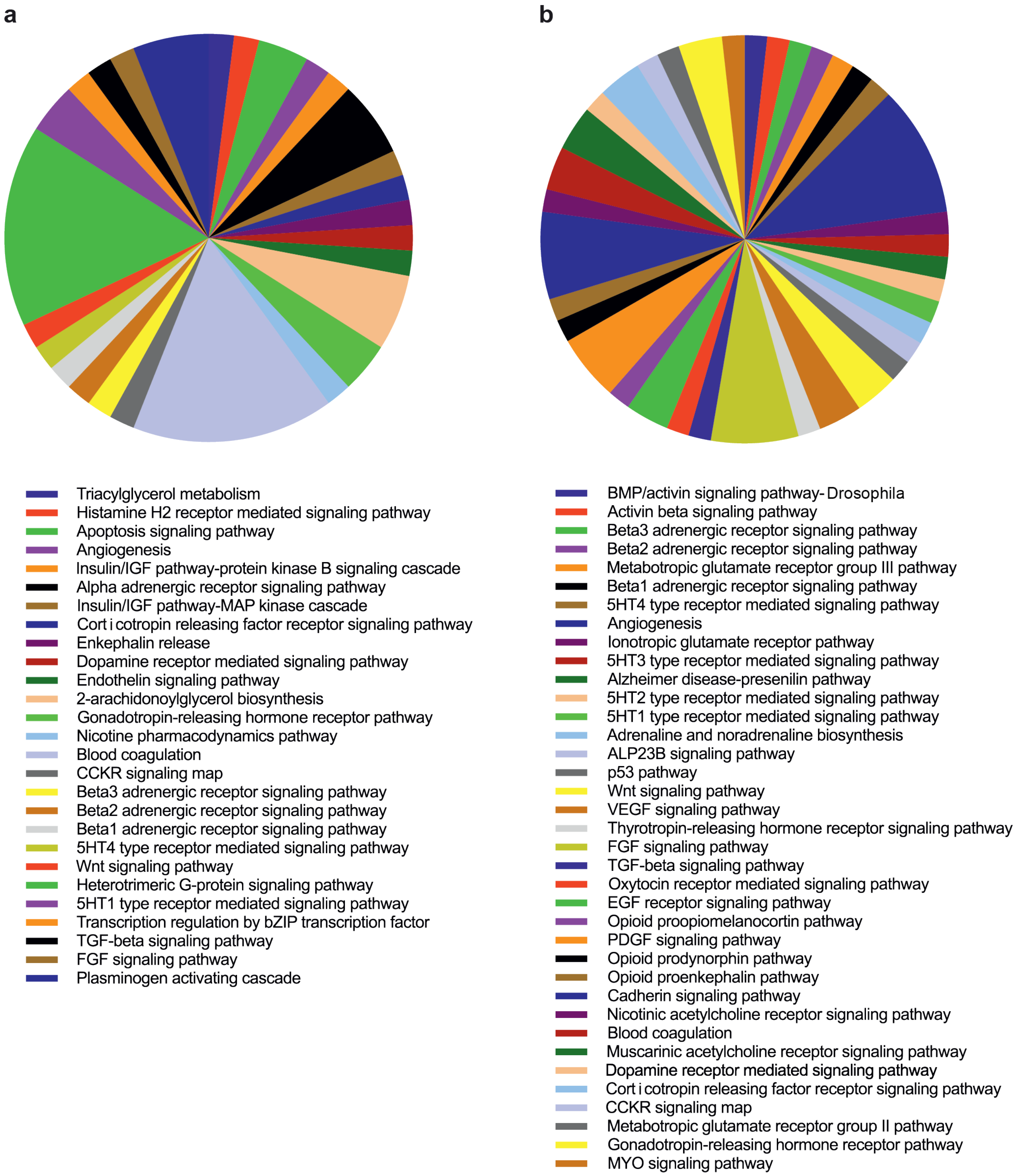

Supplementary Fig. 1. a-b Gene-ontology (GO) analysis of hits from the RNAi screen whose knockdown is associated with increased (**a**) or decreased (**b**) pupal size. Underlying data are provided in the Source Data file.

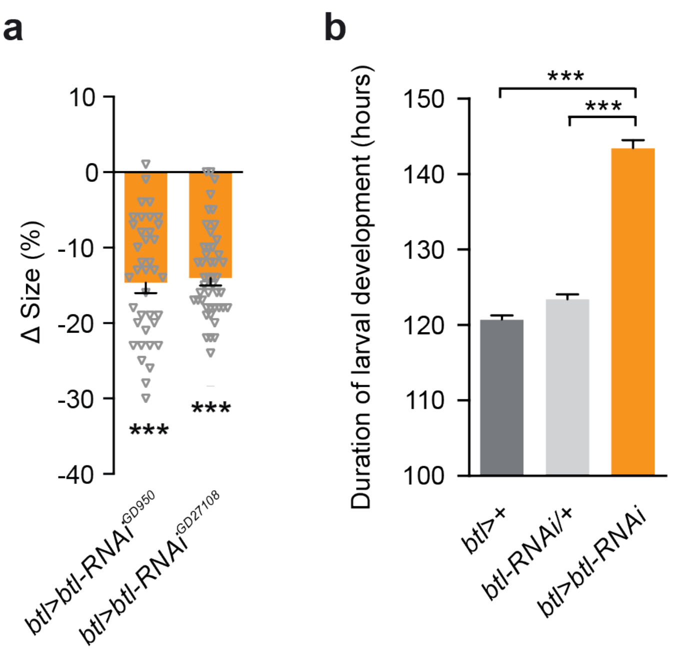

Supplementary Fig. 2. a Knockdown of *breathless* (*btl*) in the tracheae (*btl*>*btl-RNAi*) with two additional RNAi lines from VDRC (transformant ID numbers 950 and 27108) targeting independent sequences of *btl* reduces pupal body size compared to the control (*btl*> crossed to wild type, w^{1118}). *n* = 39-48. **b** Duration of larval development determined by the onset of pupariation of animals with trachea-specific *btl* knockdown compared to *btl*>+ driver (*btl*> crossed to wild type, w^{1118}) and *btl-RNAi*/+ (*btl-RNAi* crossed to wild type, w^{1118}) controls. *n* = 46-69. Statistics: one-way ANOVA with Dunnett's multiple-comparisons test. ***P<0.001, compared to the control. Error bars indicate standard error of the mean (SEM). Underlying data are provided in the Source Data file.

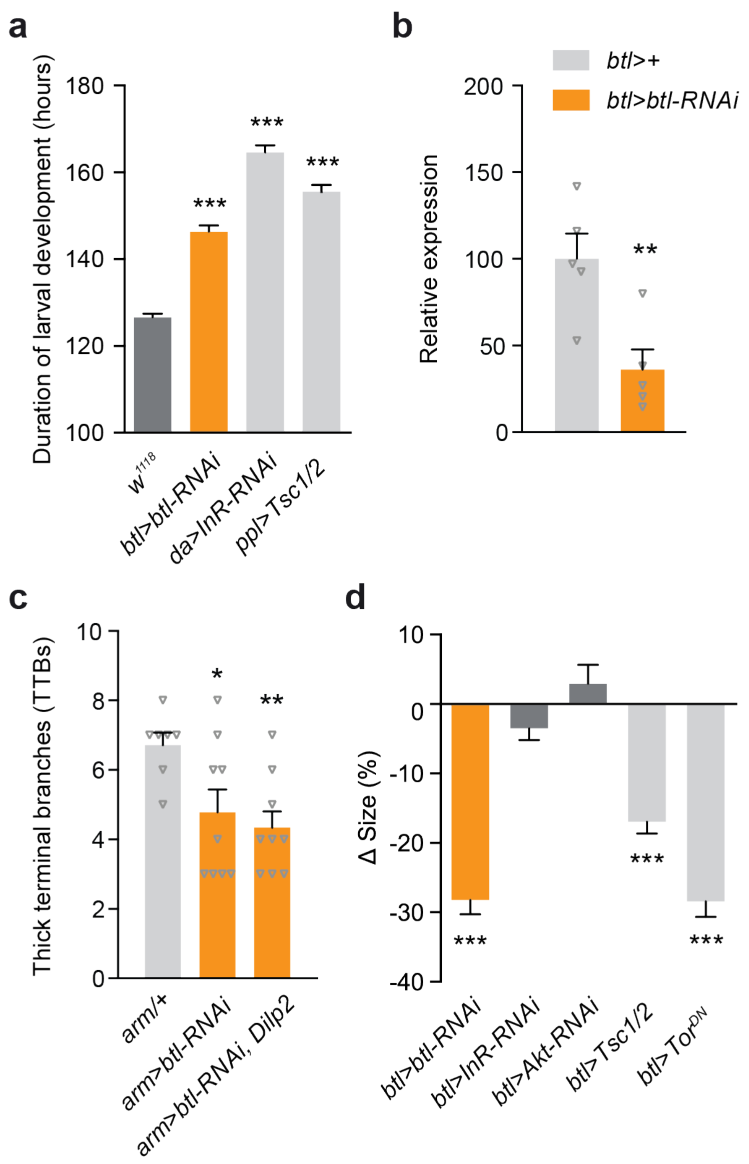

Supplementary Fig. 3. a Duration of larval development determined by the onset of pupariation of *da>InR-RNAi* animals with global knockdown of the insulin receptor (*InR*), and *ppl>Tsc1/2* animals with fat-body-specific Tor inhibition compared to controls (w^{1118}). n = 60-74. **b** Transcript levels of the ecdysone-inducible *E75B* gene. n = 5. **c** Effect of global *btl* knockdown and *Dilp2* overexpression using the weak ubiquitous *arm>* driver on the number of thick terminal branches (TTBs) per terminal cell, quantified as the number of cell projections. n = 7-9. **d** Pupal size changes in animals with trachea-specific loss of *breathless* (*btl*) (*btl>btl-RNAi*), insulin signal transduction (*btl>InR-RNAi* and *btl>Akt-RNAi*), or Tor signaling (*btl>Tsc1/2* and *btl>*Tor^{DN}) compared to *btl>+* controls (*btl>* crossed to wild type, w^{1118}). n = 22-79. Statistics: one-way ANOVA with Dunnett's test for multiple comparisons and Student's t-test for pairwise comparisons. *P<0.05, **P<0.01, ***P<0.001, compared to the control. Error bars indicate standard error of the mean (SEM). Underlying data are provided in the Source Data file.

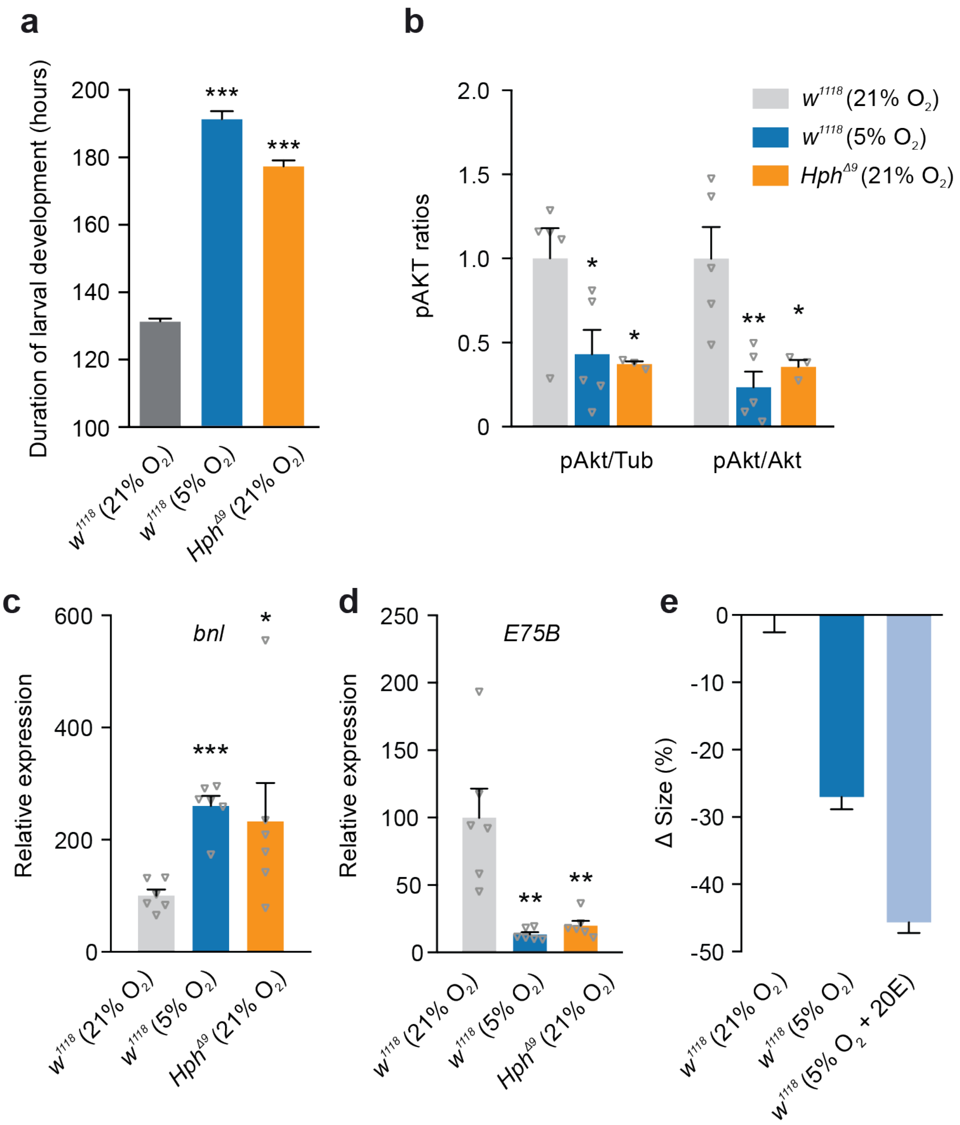

Supplementary Fig. 4. a Duration of larval development determined by the onset of pupariation of wild-type (*w[1118]*) animals under normoxia (21% O_2) and hypoxia (5% O_2), and *Hph* mutant animals under normoxia. *n* = 57-73. **b** Whole-animal levels of phosphorylated Akt (pAkt) kinase determined by immunoblotting in normoxia (21% O_2) and hypoxia (5% O_2), and *Hph* mutant animals normalized to alpha-Tubulin (Tub) or Akt levels. *n* = 3-5. **c-d** Transcript levels of *branchless* (*bnl*) (**c**) and ecdysone-inducible gene *E75B* (**d**) in whole larvae under normoxia in wild-types and *Hph* mutants and in wild-types under hypoxic conditions. *n* = 6. **e** Effects of 20-hydroxyecdysone (20E) feeding on pupal size of wild type (*w[1118]*) animals under hypoxia. *n* = 27-29. Statistics: one-way ANOVA with Dunnett's multiple-comparisons test. *P<0.05, **P<0.01, ***P<0.001, compared to the control. Error bars indicate standard error of the mean (SEM). Underlying data are provided in the Source Data file.

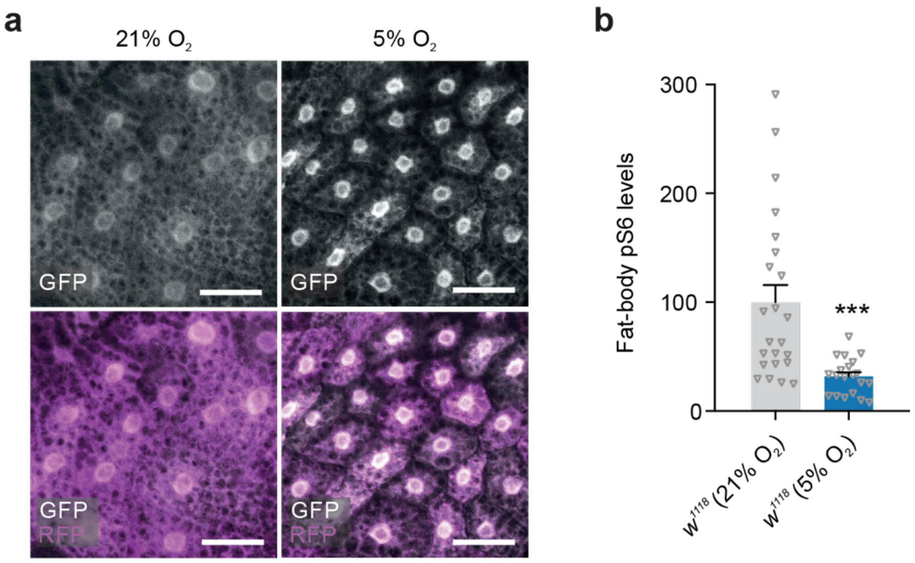

Supplementary Fig. 5. a Transgenic GFP::ODD (white) and RFP (magenta) reporter of hypoxia indicates that the fat body experiences low oxygen levels that inhibit Hph (increased GFP) when wild-type (w^{1118}) animals are incubated under 5% O_2 compared to 21% O_2 conditions. Scale bar, 20 µm. **b** Fat-body phosphorylated ribosomal protein S6 (anti-pS6) levels are reduced in larvae reared under hypoxia (5% O_2). n = 20-23. Statistics: Student's t-test for pairwise comparisons. ***P<0.001, compared to the control. Error bars indicate standard error of the mean (SEM). Underlying data are provided in the Source Data file.

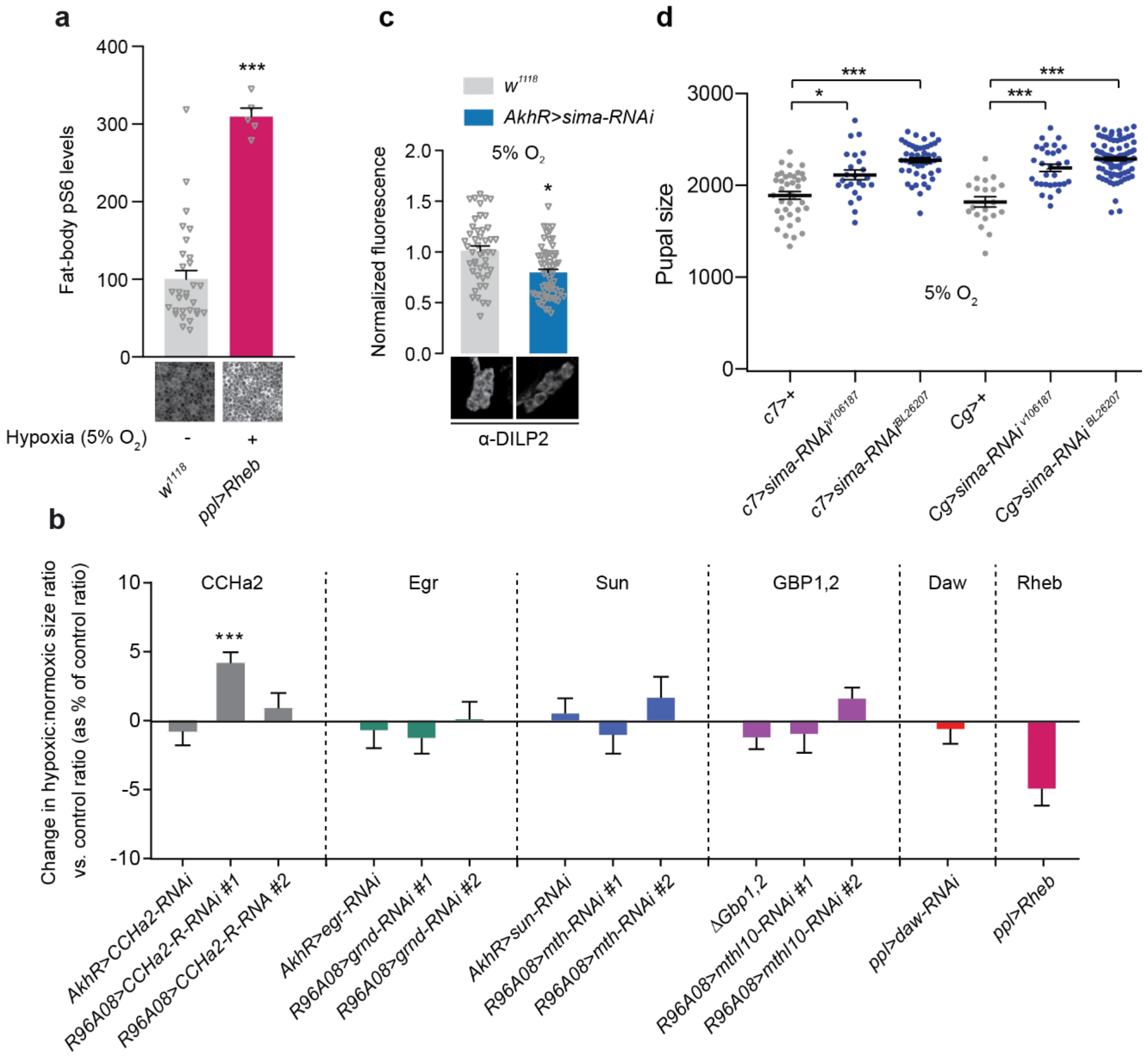

Supplementary Fig. 6. a Fat-body-specific expression of Rheb stimulates S6 phosphorylation under hypoxia, indicating increased Tor pathway activity. *n* = 5-30. **b** We conducted a mini-screen of known fat-body factors and IPC-expressed receptors for hypoxia-specific effects, hypothesizing that knocking down a factor or receptor that is involved in the insulinostatic response would rescue the size reduction (*i.e.*, lead to larger pupae) under hypoxia. *AkhR-GAL4::p65* (*AkhR>*) or *ppl-GAL4* (*ppl>*) drove RNAi against secreted factors and *Rheb* expression (inducing activation of Tor) in the fat body, while *R96A08-GAL4* (*R96A08>*) was used to knock down their respective receptors in the IPCs. Zero change indicates that an RNAi treatment had no hypoxia-specific effect – the ratio between hypoxic and normoxic sizes was the same as for controls. Roughly +20% would represent a complete blockage of hypoxia-induced size reduction. *CCHa2/CCHa2-R* results are inconsistent with each other, and a size increase upon *CCHa2-R* knockdown is inconsistent with its reported insulinotropic effects; thus perhaps the *CCHa2-R-RNAi-#1* phenotype reflects off-target effects. *n* = 1-14 vials (4-168 hypoxia:normoxia comparisons) per genotype. **c-d** Fat-body-specific *sima/HIF-1a* RNAi-mediated knockdown (mimicking the Tor-independent aspects of fat-body normoxia) reduces hypoxia-induced DILP2 retention (**c**) and partially rescues hypoxia-induced systemic body growth inhibition (**d**). c: *n* = 40-55. d: *n* =20-47. Statistics: two-tailed Student's t-test for pairwise comparisons versus controls. ***P<0.001, compared to the *Luciferase* control. Error bars indicate standard error of the mean (SEM). Underlying data are provided in the Source Data file.

Supplementary Table 1. Primers used in this study in qPCR assays.

**Target gene**	**Forward primer**	**Reverse primer**
branchless	TGCCCTATCACAGAGTTGC	ACCTACACGAACGCCATCAC
Dilp2	CTCAACGAGGTGCTGAGTATG	GAGTTATCCTCCTCCTCGAACT
Dilp3	CAACGCAATGACCAAGAGAAC	GCATCTGAACCGAACTATCACTC
Dilp5	ATGGACATGCTGAGGGTTG	GTGGTGAGATTCGGAGCTATC
Eip75B (E75B)	CAACAGCAACAACACCCAGA	CAGATCGGCACATGGCTTT
InR	CTCAGCCATACCAGGGACTTT	CTCTCCATAACACCGCCATC
RpL32	AGTATCTGATGCCCAACATCG	CAATCTCCTTGCGCTTCTTG
