# Supplementary material for: A fat-tissue sensor couples growth to oxygen availability by remotely controlling insulin secretion
Source: Nat Commun. 2019 Apr 26;10:1955. doi: 10.1038/s41467-019-09943-y (PMC6486587; doi:10.1038/s41467-019-09943-y)
Supplement: Supplementary file 3 — Description of Additional Supplementary Files [file 41467_2019_9943_MOESM3_ESM.pdf]

Description of Additional Supplementary Files

File Name: Supplementary Data 1
Description: RNAi screening data. Columns show VDRC or Bloomington stock ID numbers, CG numbers, gene names, gene abbreviation symbols, the stock center from which the transgenic lines were obtained, n = the number of pupae in the sample, the mean pupal size, the Z score for the sample, the percentage difference of the sample from the mean of the entire data set, and the number of standard errors away from this mean the sample fell
